# Automated System to Capture Patient Symptoms From Multitype Japanese Clinical Texts: Retrospective Study

**DOI:** 10.2196/58977

**Published:** 2024-09-24

**Authors:** Tomohiro Nishiyama, Ayane Yamaguchi, Peitao Han, Lis Weiji Kanashiro Pereira, Yuka Otsuki, Gabriel Herman Bernardim Andrade, Noriko Kudo, Shuntaro Yada, Shoko Wakamiya, Eiji Aramaki, Masahiro Takada, Masakazu Toi

**Affiliations:** 1 Department of Information Science Nara Institute of Science and Technology Ikoma Japan; 2 Graduate School of Medicine Kyoto University Kyoto Japan; 3 Center for Information and Neural Networks Advanced ICT Research Institute Osaka Japan; 4 Department of Breast Surgery Kansai Medical University Hirakata Japan; 5 Tokyo Metropolitan Cancer and Infectious Disease Center Komagome Hospital Tokyo Japan

**Keywords:** natural language processing, named entity recognition, adverse drug reaction, adverse event, peripheral neuropathy, NLP, symptoms, symptom, machine learning, ML, drug, drugs, pharmacology, pharmacotherapy, pharmaceutic, pharmaceutics, pharmaceuticals, pharmaceutical, medication, medications, adverse, neuropathy, cancer, oncology, text, texts, textual, note, notes, report, reports, EHR, EHRs, record, records, detect, detection, detecting

## Abstract

**Background:**

Natural language processing (NLP) techniques can be used to analyze large amounts of electronic health record texts, which encompasses various types of patient information such as quality of life, effectiveness of treatments, and adverse drug event (ADE) signals. As different aspects of a patient’s status are stored in different types of documents, we propose an NLP system capable of processing 6 types of documents: physician progress notes, discharge summaries, radiology reports, radioisotope reports, nursing records, and pharmacist progress notes.

**Objective:**

This study aimed to investigate the system’s performance in detecting ADEs by evaluating the results from multitype texts. The main objective is to detect adverse events accurately using an NLP system.

**Methods:**

We used data written in Japanese from 2289 patients with breast cancer, including medication data, physician progress notes, discharge summaries, radiology reports, radioisotope reports, nursing records, and pharmacist progress notes. Our system performs 3 processes: named entity recognition, normalization of symptoms, and aggregation of multiple types of documents from multiple patients. Among all patients with breast cancer, 103 and 112 with peripheral neuropathy (PN) received paclitaxel or docetaxel, respectively. We evaluate the utility of using multiple types of documents by correlation coefficient and regression analysis to compare their performance with each single type of document. All evaluations of detection rates with our system are performed 30 days after drug administration.

**Results:**

Our system underestimates by 13.3 percentage points (74.0%−60.7%), as the incidence of paclitaxel-induced PN was 60.7%, compared with 74.0% in the previous research based on manual extraction. The Pearson correlation coefficient between the manual extraction and system results was 0.87 Although the pharmacist progress notes had the highest detection rate among each type of document, the rate did not match the performance using all documents. The estimated median duration of PN with paclitaxel was 92 days, whereas the previously reported median duration of PN with paclitaxel was 727 days. The number of events detected in each document was highest in the physician’s progress notes, followed by the pharmacist’s and nursing records.

**Conclusions:**

Considering the inherent cost that requires constant monitoring of the patient’s condition, such as the treatment of PN, our system has a significant advantage in that it can immediately estimate the treatment duration without fine-tuning a new NLP model. Leveraging multitype documents is better than using single-type documents to improve detection performance. Although the onset time estimation was relatively accurate, the duration might have been influenced by the length of the data follow-up period. The results suggest that our method using various types of data can detect more ADEs from clinical documents.

## Introduction

Processing large amounts of data using artificial intelligence can help to rapidly obtain a comprehensive understanding of the patient status, which can potentially streamline medical studies focusing on patient stratification, drug safety, and adverse drug event (ADE) detection. Particularly, information on ADEs must be collected prospectively, which is expensive and time-consuming. Even when data are collected retrospectively from electronic health records containing information on various modalities, it is challenging to comprehensively survey the medical details of a large number of patients.

Fortunately, natural language processing (NLP) methods can be used to aid such tasks. Recent advances in NLP have enabled the automatic extraction of contextual information from text. Bidirectional Encoder Representations from Transformers (BERT), a transformer-based model released in 2019, has achieved high accuracy in many NLP tasks [1]. Particularly, using diverse medical records for training machine learning models on multiple aspects of patient information can improve their prediction accuracy in the medical domain, leading to the development of specialized models such as ClinicalBERT and BioBERT [2-5].

The ADE detection systems that use such models have been applied to actual texts in existing research [6-10]. Several studies have also used NLP in retrospective observational studies, similar to the approach used in this study [11,12]. McKenzie et al [11] conducted a retrospective analysis of pneumonia using electronic health records; however, they used rule-based NLP methods for 2 types of documents, clinical notes and radiology reports written by physicians, thus leaving room for further investigation into performance.

In addition to physicians’ records, medical institutions have a wide variety of documents from multiple co-medical personnel, including nursing records, pharmacists’ progress notes, and medication orders. Using multiple types of medical documents on retrospective studies requires a comprehensive and robust data analysis because of the expected decrease in missing event detection. While such an analysis is difficult when performed manually due to time requirements and human resource constraints, an NLP-based approach should be more efficient and effective.

A common method for information extraction using NLP is to treat it as a text classification task specific to each document type. However, document-specific text fine-tuning requires that each model be fine-tuned individually for each specific document type, which does not fully demonstrate the strength of automated processing. Fine-tuning a model requires labeled data, and since such data are unlikely to be available beforehand, it requires manual annotation by health care professionals. Even if annotated data are available, privacy concerns and data security restrictions imposed by medical institutions usually make access to them rather difficult. Furthermore, as the transmission of such data over the internet is usually not allowed, the usage of the cloud computational power becomes unfeasible.

For such reasons, fine-tuning models for each individual document type becomes impractical. Therefore, in this study, we used a named entity recognition (NER) model for medical documents, which does not require fine-tuning for each document type. The NER model can be easily used for information extraction without fine-tuning with the target documents since it is already fine-tuned with medical documents to detect symptoms.

**Figure 1:**
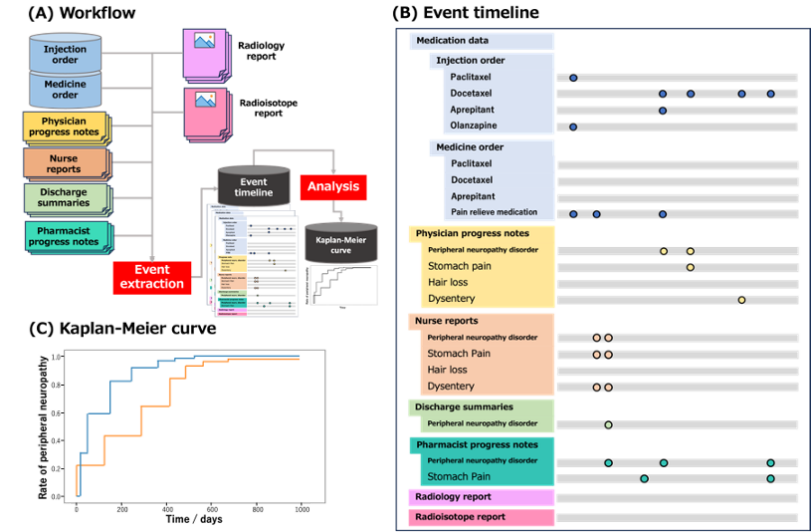
Data flow of the proposed system. (A) shows the events from multiple types of documents are extracted. An event timeline (B) is created from each clinical data using the natural language processing method, and then the curve (C) is created based on the aggregated results. The dots in the event timeline indicate the timing at which the description of drug administration or symptom onset is recorded. Based on (B), patients who received the target drug (a taxane drug in this study) are selected, and the Kaplan-Meier curve (C) is generated.

In this study, we examined the usefulness of analyzing various medical Japanese documents, including medical records written by physicians and co-medical professionals, to capture the onset and duration of ADEs. Figure 1 shows the basic idea of our approach. Our medical NLP method aims to comprehensively analyze ADE-relevant information contained in medical documents, including nursing records, pharmacist progress notes, and other medical texts, in addition to physicians’ records. Our method identified more ADEs from various document types compared to a single type, resulting in a performance similar to that of the typical manual analysis.

## Methods

### Overview

Our study uses a retrospective observational approach based on NLP, which enables the handling of a large amount of data. The NLP techniques used were NER and normalization, which extract symptoms from documents and transform them into their standardized forms. We evaluated our method by obtaining the Kaplan-Meier curves based on symptoms that were normalized to peripheral neuropathy (PN). In addition, we also evaluated the duration of PN.

### Materials

This study used data from all patients diagnosed with breast cancer (N=2289) treated at the Kyoto University Hospital between 2019 and 2021. The patient data consisted of 2 types of medication orders (structured data) and 6 types of texts written in Japanese (unstructured data). We apply NLP methods to extract information from such unstructured data. Unstructured data require an NLP method to extract information, such as ADEs. Table 1 lists the number of all breast cancer patient orders and text data records included in each document.

**Table 1:** Amount of order data and text data. The unit of record is per drug for order data and per timing recorded by physicians or co-medicals for text data.

Data type		Records, n
**Order data**		
	Injection order	44,896
	Medicine order	63,077
**Text data**		
	Physician progress notes	159,736
	Nursing records	40,385
	Discharge summaries	23,073
	Radiology reports	5663
	Radioisotope reports	1147
	Pharmacist progress notes	29,148

### Inclusion and Exclusion Criteria

The aim of this study was to leverage the strengths of NLP to automatically analyze a large number of documents and evaluate the usefulness of the proposed method. We selected PN as our disease for evaluation, as it satisfies the following conditions: (1) side effects are long-lasting, which means that monitoring many documents is required, and (2) information on the onset of the symptom is not normally included in structural databases.

**Figure 2:**
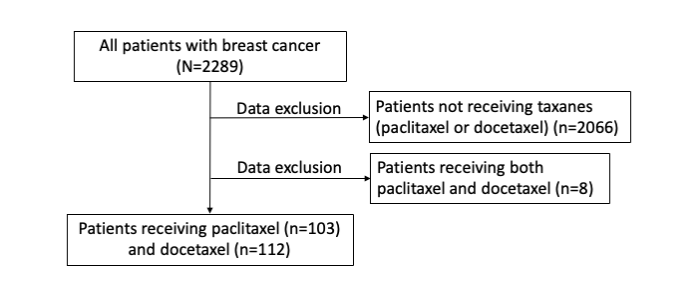
Flowchart describing the procedure for selecting patient data according to criteria.

We selected taxane drugs, such as paclitaxel and docetaxel for the evaluation, as they frequently cause PN. As shown in Figure 2, patients receiving either type of taxane drugs were included in the analysis, whereas those receiving both paclitaxel and docetaxel were excluded. Patients selected according to these criteria were then analyzed for the development of PN as an outcome.

Patients who received both drugs were excluded to prevent them from introducing noise in the analysis of PN onset and duration. Administering a different taxane drug during monitoring sessions, which had not been given previously, could have adversely affected the study results.

### Comparison With the Kaplan-Meier Curves

**Figure 3:**
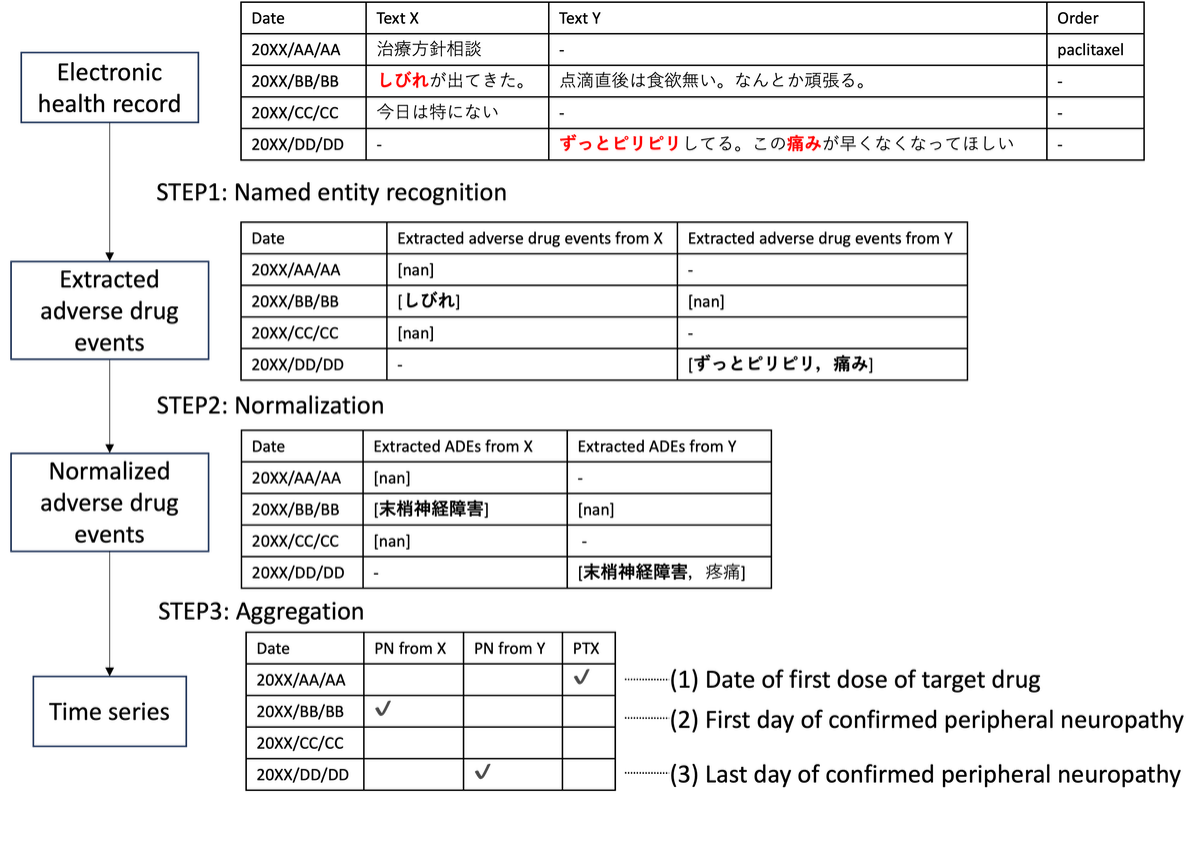
Workflow of our natural language processing system, which is composed of named entity recognition, normalization, and aggregation. Text X and Text Y are examples of 2 types of documents respectively, for example, physician progress notes and pharmacist progress notes.

Using information extracted from multitype texts by applying our NLP method, we measured the number of days until the onset of PN after the administration of taxane drugs. As shown in Figure 3, our system is composed of 3 steps: entity recognition, normalization, and aggregation. We compared these results with those reported manually in a previous report [13].

### Named Entity Recognition

The data required for this study, which included the dates on which the symptoms occurred (obtained from text data) and drugs that were administered (obtained from medication orders), were obtained as follows: to obtain symptom data, we applied NER, an NLP method that recognizes and extracts mentions of named entities in text. We used this method to identify symptoms related to PN. We adopted MedNER-CR-JA, which is a BERT-based NER model trained using Japanese case reports [14]. Since BERT can only process a maximum of 512 tokens at a time, sentences were separated by line breaks. Only the symptoms with positive factuality, as extracted by the model, were used in the analysis.

### Normalization

The extracted entities are normalized by Levenshtein distance matching using a disease name dictionary (MedDic-CANCER-ADE-JA) [15]. This dictionary contains surface forms and normalized forms with respect to the side effects of anticancer drugs. We select the dictionary surface form that has the lowest Levenshtein distance in relation to the extracted term and then convert it to the related normalized form [16]. The code for this step including NER can be accessed through GitHub [17].

### Aggregation

**Figure 4:**
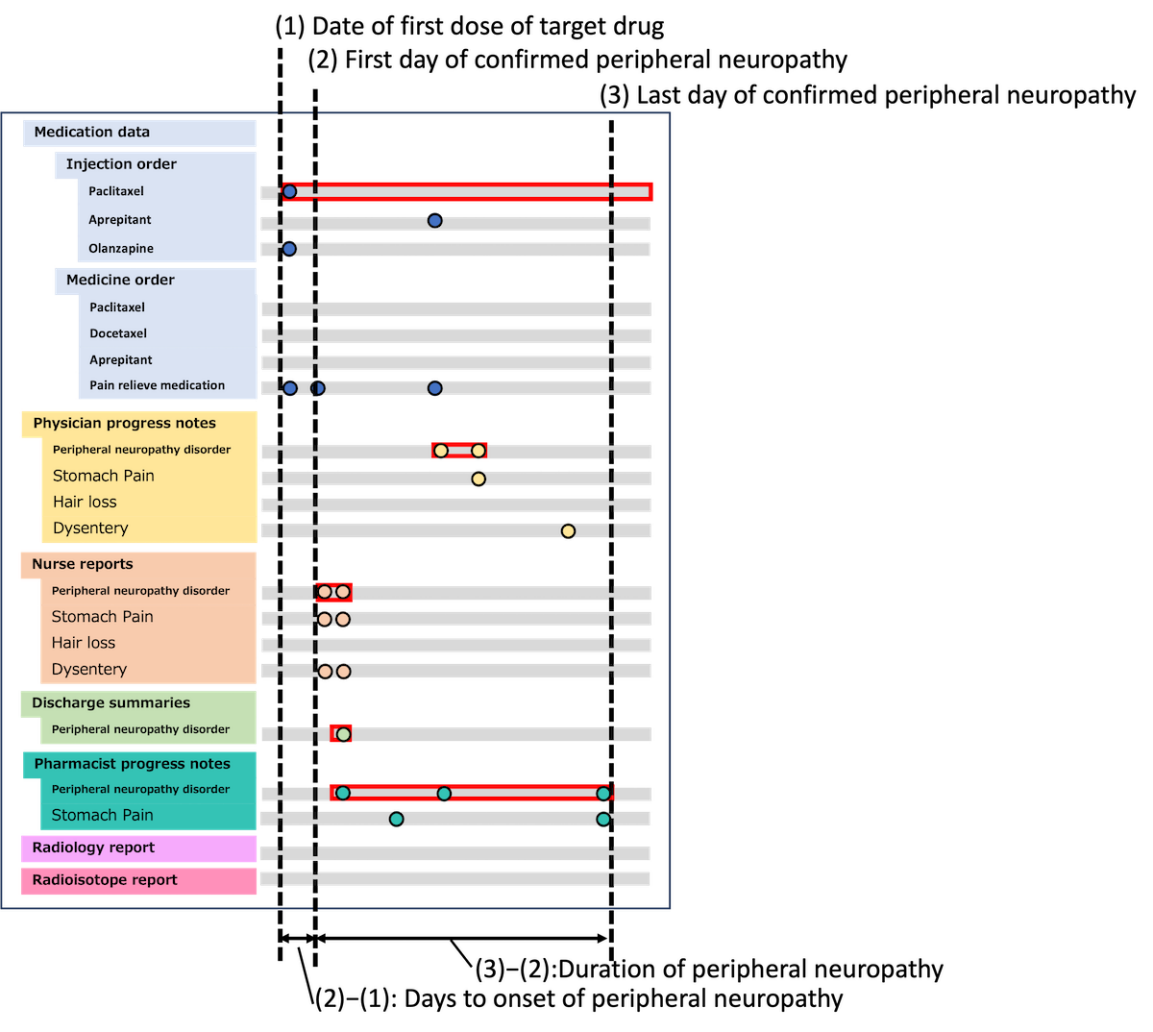
Event timeline from multiple types of data and calculation of the number of days of peripheral neuropathy onset and duration.

We focused on the expression normalized to PN and conducted the analysis among the converted terms. Specifically, the onset date of PN was defined as the first date on which the expression was normalized to PN in any type of document. The cumulative percentage of patients who developed PN was calculated along the time series. As shown in Figure 4, the onset date was the number of days since the first dose of paclitaxel or docetaxel. We defined the period of residual PN as the period up to the date on which the expression normalized to PN was last identified. The onset date and residual duration for each patient were summed to obtain a Kaplan-Meier plot of onset timing or residual duration, respectively. The onset date and residual duration of each patient were aggregated to obtain a Kaplan-Meier plot of onset timing or residual duration, respectively.

We propose that this definition would be more robust if the system analyzed various types of documents reviewed by multiple medical personnel. Increasing the diversity of documents analyzed reduces the risk of overlooking symptoms.

### Evaluation

The cumulative percentage of the patients’ PN is displayed using the event date on which PN was first identified.

We compared the results produced by our NLP system (Paclitaxel_NLP) with previous results obtained by manual extraction (Paclitaxel_MAN) based on the percentage of PN at 30 days. The detection rate was evaluated by subtracting the percentage of detections achieved by our system from the percentage of detections obtained through manual extraction [13]. We focused on the incidence of PN at 30 days since most patients generally develop the disease after 30 days [13].

In addition, the Pearson correlation coefficient was calculated for the 2 types of paclitaxel results from our system and manual results up to 101 days, the maximum duration in the previous report.

In addition, multiple regression analysis was performed to analyze the results calculated using all records and the results from each record to evaluate which explanatory variables had a greater impact.

### Ethical Considerations

This study, which was evaluated and approved by the ethics committee of Kyoto University Graduate School and Faculty of Medicine, Japan (R3723-2), was performed in compliance with the Declaration of Helsinki.

## Results

### Preliminary Result

As shown in Figure 2, among the 2289 patients from the data set, 215 were selected (paclitaxel, n=103; docetaxel, n=112). A total of 2066 patients who did not receive paclitaxel nor docetaxel and 8 patients who received both paclitaxel and docetaxel were excluded. The median age of the participants was 59 years (range 33-78) for the paclitaxel-treated patients and 52 (25-73) years for the docetaxel-treated patients, which is not significantly different from the median age of 53 (range 22-70) years in previous reports. The mean and maximum follow-up periods were, respectively, 380.3 and 1264 days for paclitaxel-treated patients and 545.1 and 1080 days for docetaxel-treated patients.

A total of 7428 symptom expressions were extracted (paclitaxel=3732 and docetaxel=3696), of which 5057 (paclitaxel=2804 and docetaxel=2253) were positive for symptom factuality and 879 (paclitaxel=569 and docetaxel=310) were PN-related.

**Figure 5:**
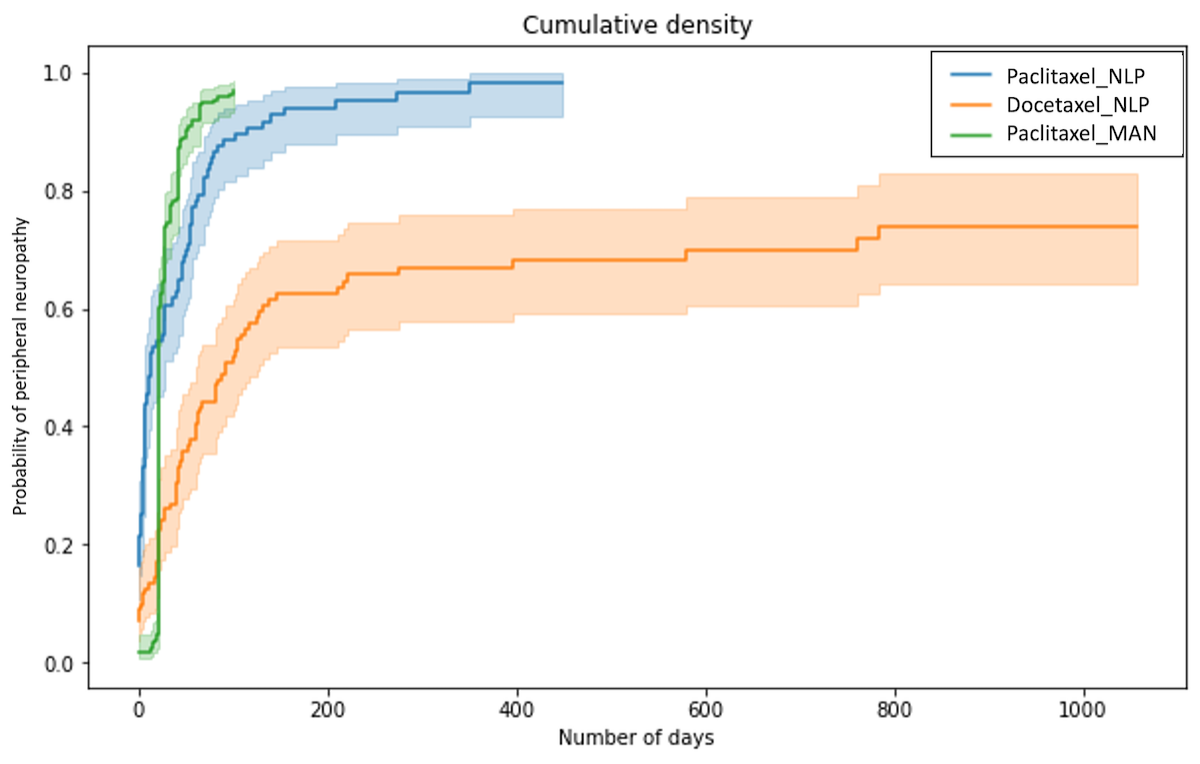
Kaplan-Meier curves of the results obtained by our system (Paclitaxel_NLP and Docetaxel_NLP) and the previous results obtained using a manual method (Paclitaxel_MAN). The solid line indicates the proportion of patients who developed peripheral neuropathy among those who received paclitaxel or docetaxel. Filled areas indicate 95% CIs.

Figure 5 shows the Kaplan-Meier curves of the results obtained by our system and the previous results obtained using a manual method. Of the 103 patients who received paclitaxel (n=103), 97 had confirmed PN; from the 112 patients who received docetaxel (n=112), 76 had confirmed PN.

### Comparison With the Kaplan-Meier Curves

The incidence of PN caused by paclitaxel was 60.7% at 30 days, and as the previous research reported incidence was 74% at 30 days, and the detection gap was 13.3 points (74.0%-60.7%) [13]. The percentages represent the proportions of patients who were determined to have developed PN from documents.

The result does not entirely reflect the actual onset of the disease; however, the system detected PN in almost all patients over 1 year, which seems accurate enough. The correlation coefficient between the results obtained by our system (Paclitaxel_NLP) and those obtained manually (Paclitaxel_MAN) was 0.87, with a *P* value of 1.72×10^–32^ (<.05), indicating a high correlation.

**Figure 6:**
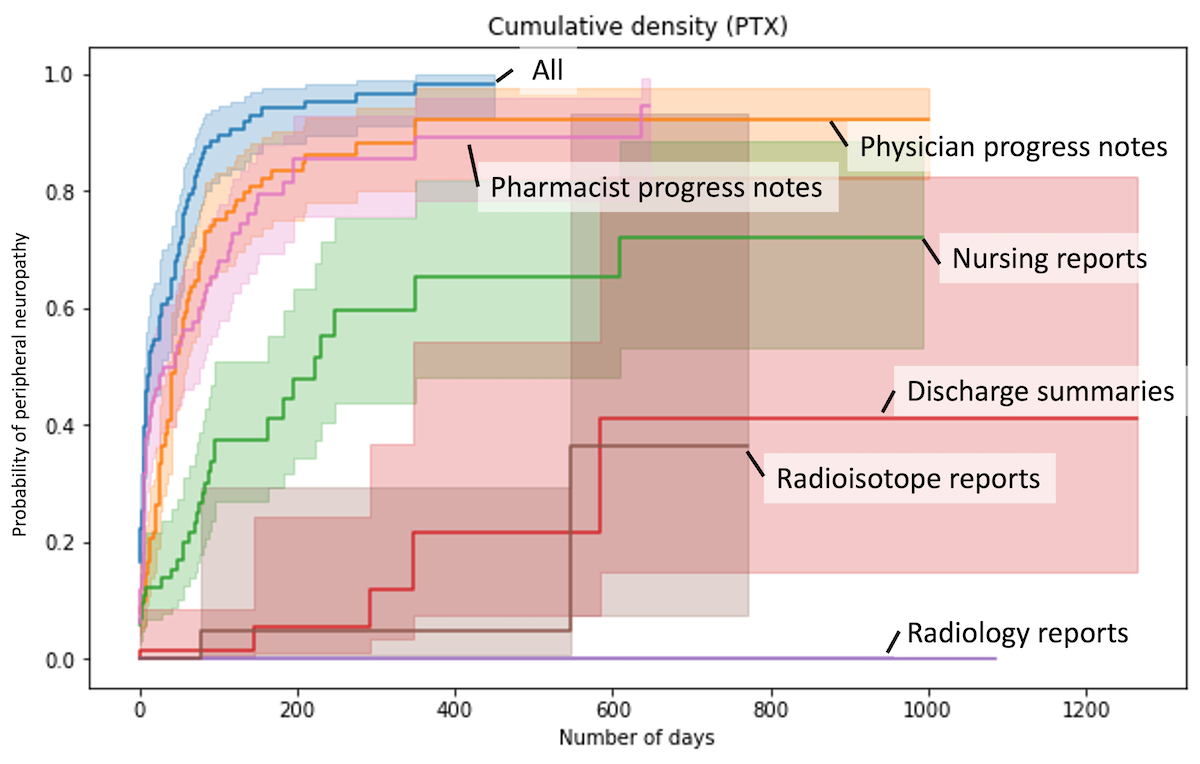
Comparison between the results from each document type and all document types.

Figure 6 shows the comparison between the results from per document type and all document types. The percentages of PN identified in each document type, in descending order, were physician progress notes, pharmacist progress notes, nursing records, discharge summaries, radioisotope reports, and radiology reports.

In order to assess which documents influenced the results calculated from all documents, multiple regression analyses were performed. The results from all documents were used as predictor variables, and the results from each document as explanatory variables. The respective regression coefficients and SD (shown in parentheses) were 0.70 (0.04) for pharmacist progress notes, 0.35 (0.03) for physician progress notes, 0.32 (0.09) for nursing records, 1.39 (1.67) for discharge summaries, 1.64×10^–16^ (1.64×10^–16^) for radiology reports, and –0.53 (0.21) for radioisotope reports. The results suggest the importance of physician progress notes, pharmacist progress notes, and nursing reports among the document types.

When all records were used, the system was able to detect the onset of all PNs that could be detected at 350 days. On the other hand, the same results could not be obtained, even after 600 days, when each type of documents was used independently.

The results from the pharmacist records were similar to those from all types of documents in the initial period but remained almost constant after 200 days, with few new cases of PN detected. Nursing records, which contain many records of patient care, were expected to be effective in detecting adverse drug reactions such as PN, but the detection rate was less than half that of physician and pharmacist records. The detection rate for discharge summaries, radiology reports, and radioisotope reports was very low (less than half), suggesting that these types of documents are less useful for the target diseases and target drugs in this study.

**Figure 7:**
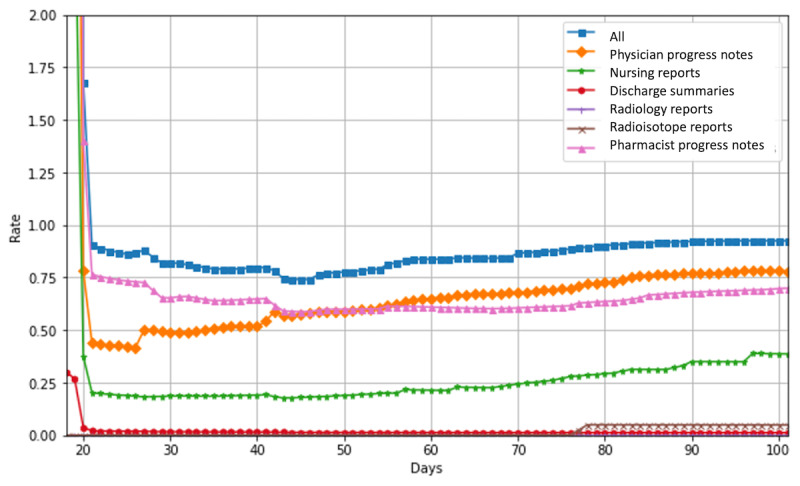
Rates of patients with peripheral neuropathy detected in each document compared with manual results.

Figure 7 shows the detection rate of patients with PN in each document compared with the manual results. At 30 days, the detection rate compared with manual was 65.3% for pharmacist progress notes, 49.1% for physician progress notes, 18.6% for nursing records, 1.6% for discharge summaries, 0% for radioisotope reports, and 0% for radiology reports. The detection rates of all records were lower than the combined detection rate of all records (82.0%). This suggests that the use of multiple types of documents is effective.

In the early stage of the observation period, automatic extraction tended to overdetect PN. This is likely due to the incorrect detection of expressions related to side effect descriptions, which will be discussed in the error analysis section. The detection rate decreases in the middle of the period and slowly increases in the latter half. In the first half of the observation period, pharmacist progress notes showed the highest performance in detecting the results from a single type of documents, while physician progress notes showed the highest performance in the second half of the observation period. It is interesting to note that different document types tend to have different detection rates depending on the time of observation.

## Discussion

### Principal Findings

In contrast to the rapid increase in the number of patients developing PN 20 days after beginning treatment in a previous report, our system detected PN at an earlier treatment stage [15].

Our method can extract symptoms from text, determine factuality, and chronologically monitor the patient’s symptoms. Therefore, as long as the target symptoms are described in the text, the same method can be applied to any symptom and all drugs other than taxanes, making it a versatile and scalable method. Although there is still room for improvement in accuracy, the analysis can be automated to reduce research costs, particularly in observational studies, where large amounts of text need to be analyzed.

### Error Analysis

The detection rate by our system may be affected by false negatives, suggesting that the model overlooks expressions that are difficult to detect, such as onomatopoeic expressions, as we will discuss in this section.

A detailed analysis categorized 3 types of errors, as shown in Table 2, namely, errors in symptom extraction, factuality determination, and normalization. Among these, errors in factuality determination and errors in normalization were found to increase the likelihood of outputting false positives. An example of errors in factuality determination is that explanations such as “this medicine has a risk of PN” can be misinterpreted as PN. Normalization errors included instances of normalizing expressions not limited to PN, such as “numbness” in “Numbness + in upper extremities due to cervical stenosis” to PN. All 3 types of errors were identified as false negatives. As a symptom extraction error, it was confirmed that the onomatopoeic “tingling (ビリビリ, biribiri)” was not extracted. The NER model is not effective at recognizing more informal expressions, such as onomatopoeias, probably because the model is fine-tuned using case reports, which are relatively formal sentences. As for errors in factuality determination, “tingling (ぴりぴり, piripiri)” was not extracted in “Even a rest does not stop tingling sensation in my hands.” Although this text implies the positive factuality of the symptoms, the presence of negation in the sentence may have interfered with the model’s determination of factuality. Note that this expression is onomatopoeic and translated into the same word in English. However, this is a different expression in Japanese, and the symptoms were properly extracted. Other expressions such as “There is a risk of paralysis (麻痺のリスクあり)” and “Explained side effects of eribulin…PN… (エリブリンの副作用...末梢神経障害...について説明)” were also incorrectly extracted. This is because such expressions are rarely used in case reports.

**Table 2:** Types of errors in the detection of peripheral neuropathy. Original Japanese texts are in parentheses. Italicized text is the expression extracted by named entity recognition.

Types of errors and examples			Prediction outcome
**Errors in extracting symptoms**			
	Have a tingling sensation in my limbs. （手足がぴりぴりする）	False negative	
**Errors of factuality determination**			
	Explained side effects of eribulin: decreased blood counts, risk of infection, *PN*, fatigue, decreased appetite, etc. （エリブリンの副作用：血球減少、感染のリスク、*末梢神経障害*、倦怠感、食欲低下等について説明）	False positive	
	Owing to fractured thoracic vertebrae, there is a risk of paralysis during rehabilitation （リハビリは胸椎が骨折で*麻痺*のリスクあり）	False positive	
	Even a rest does not stop tingling sensation in my hands. （手の*びりびり*は休んでもマシにはなりません。）	False negative	
**Errors of normalization**			
	*Numbness* + in upper extremities due to cervical stenosis. （頚椎狭窄で上肢に*しびれ*＋）	False positive	
	*Cellulitis of the right upper extremity* （*右上肢蜂窩織炎*）	False positive	
	*No abnormal changes* of note in blood sampling results（特記すべき*異常変化*を採血結果に認めない）	False positive	
	Bilateral supraclavicular lymph nodes, mediastinal lymph nodes, and para-aortic lymph node metastases are considered to be affected, *decreased accumulation* （両側鎖骨上リンパ節、縦隔リンパ節、傍大動脈リンパ節転移は効果ありと考えられる*集積低下*）	False positive	
	After wearing a supporter, edema got better, but *pain and numbness* appeared. （サポーターをしたら、浮腫は良くなったが、逆に*痛み・しびれ*が出てきた。）	False negative	

As a normalization error, the expression such as “pain and numbness” in the sentence “After wearing a supporter, edema got better, but pain and numbness appeared.” was normalized incorrectly because the model extracted not only numbness but also pain and numbness as a coherent expression, and any surface terms in the dictionary did not match sufficiently in this case. False positives in normalization are influenced by the surface form of the dictionary used. “Abnormal change (異常変化)” is matched to “sensory abnormality (感覚異常)” in the dictionary, and “hypoaccumulation (集積低下)” is matched to “hypoalgesia (痛覚低下).”Adjustment of the Levenshtein distance threshold may yield better results.

The false positive result suggests an early overdetection of PN in the automatic detection system, while the false negative result is associated with a decrease in the detection rate in the middle of the graph. As shown in Figure 7, for false negatives, our method of using multiple types of documents compensates for the lower detection rate compared with the use of a single document.

The impact of the error on clinical outcomes is that a false negative in the extracting symptoms and factuality determination represents a significant clinical risk because it means that an adverse drug reaction was missed. However, our method of using multiple types of documents reduces this risk compared with using only 1 type of document because the multiple types of documents complement each other and reduce false negatives. In the case of a false positive, the risk of adverse drug reactions is overestimated, and the patient may not be able to choose an appropriate treatment if the adverse drug reaction is a factor in the drug selection decision. In addition, the same phenomenon may occur in the case of normalization errors. The linking of different symptoms may also lead to incorrect conclusions about adverse drug reactions because the symptoms that occur cannot be accurately captured. For example, an unrelated symptom may be detected as a risk, resulting in unnecessary investigations.

### Documents Containing Adverse Drug Event Information

Table 3 shows a breakdown of the number of documents and patients with PN detected in each document. Since large counts of PN detection are seen in nursing records, pharmacist progress notes, and physician progress notes, we assert that analyzing multiple types of documents, such as nursing records and pharmacist progress notes, is as important as physician progress notes. It can be inferred from these results that combining multiple types of medical documents not only enables the detection of more patient events, but also reduces the number of missed events per patient.

**Table 3:** Counts in each document type.

Document type	Documents, n				Patients, n		
	Total	Paclitaxel	Docetaxel	Total		Paclitaxel	Docetaxel
Physician progress notes	373	246	127	146		85	61
Nursing records	189	117	72	80		49	31
Discharge summaries	24	10	14	22		9	13
Radiology reports	0	0	0	0		0	0
Radioisotope reports	2	2	0	2		2	0
Pharmacist progress notes	291	194	97	137		81	56

### Duration of Adverse Drug Event

Duration of PN was calculated as the period from the date of onset to the date of the last PN detection. The median number of days of PN onset by paclitaxel was 12 days, the median number of days of last confirmed onset was 126 days from the start of administration, and the median duration was 92 days. The median number of days of PN onset by docetaxel was 45.5 days, the mean number of last observed days was 135.5 days from the start of administration, and the median duration was 64.0 days. The median duration of PN with paclitaxel reported previously was 727 days, and the results are likely to significantly underestimate the duration because of the nature of the follow-up period of the analyzed data in this study, which was approximately 1000 days at most [13].

### Limitations

The results obtained with our method are dependent on the accuracy of the NER model used. Although our model achieved the best performance in a shared task, there is still room for improvement, with an *F*_1_-score of 62.9% for the extraction performance of the relevant tags in this task [18]. This model was fine-tuned based on case reports; however, we expect that fine-tuning using annotated data from the same type of documents as those used in this study, such as nursing records and progress notes, will improve the results. In addition, dictionary matching using the Levenshtein distance is performed for normalization. The normalization may have introduced false positives and false negatives.

The onset of PN was defined as the date when PN was first identified in the text. Therefore, if a PN that occurred in the past is mentioned in the text, it is possible that the onset of PN is assessed late. Similarly, the end of the PN disease period was defined as the date on which PN was last identified. The maximum follow-up period of the studies used in this study was approximately 1000 days, which may be an underestimate of the residual duration of PN.

This method focuses on the presence or absence of PN and does not quantitatively evaluate the common terminology criteria for adverse events grade. Although this model determines only the factuality of the symptoms, a more detailed analysis can be conducted by creating a model that determines the grade.

### Conclusions

We proposed a system to detect PN by using NLP methods to allow the analysis of multityped documents automatically and concurrently. Analyses were performed on breast cancer patients receiving paclitaxel and docetaxel. As a result, many PN events were extracted from the nursing records and pharmacists’ progress notes as well as physicians’ progress notes. This approach is reasonable when considering the multiple types of records used in this study since leveraging multitype documents is better than single-type documents to improve detection performance. Based on the timing of the onset, our system underestimates by 13.3 percentage points.

We also examined persistent PN using a similar approach. Compared with the manual results, it was suggested that the duration of PN was underestimated; however, this may be due to the large difference in the follow-up periods.

Although the accuracy of the system requires further investigation, we believe that our NLP system has great potential to provide an immediate estimate of the persistence of ADEs, which traditionally requires continuous investigation and incurs high costs.

## Abbreviations

ADE: adverse drug event
BERT: Bidirectional encoder representations from transformers
NER: named entity recognition
NLP: natural language processing
PN: peripheral neuropathy
